# Intestinal electrical stimulation attenuates hyperglycemia and prevents loss of pancreatic β cells in type 2 diabetic Goto–Kakizaki rats

**DOI:** 10.1038/s41387-019-0072-2

**Published:** 2019-02-06

**Authors:** Xiaojun Ouyang, Shiying Li, Yan Tan, Lin Lin, Jieyun Yin, Jiande D. Z. Chen

**Affiliations:** 1 Veterans Research and Education Foundation, VA Medical Center, Oklahoma City, OK USA; 2 Division of Geriatrics and Gerontology, Geriatric Hospital of Nanjing Medical University, Nanjing, Jiangsu China; 3 Division of Gastroenterology and Hepatology, Johns Hopkins University, Baltimore, MD USA; 4 Division of Gastroenterology, The First Affiliated Hospital of Hainan Medical University, Haikou, Hainan China; 5 Division of Gastroenterology, The First Affiliated Hospital of Nanjing Medical University, Nanjing, Jiangsu China

## Abstract

**Background/Objective:**

Recently, intestinal electrical stimulation (IES) has been reported to result in weight loss; however, it is unclear whether it has a therapeutic potential for diabetes. The aim of the present study was to explore the potential hypoglycemic effects of IES and its possible mechanisms involving β cells in diabetic rats.

**Subjects/Methods:**

Diabetic Goto–Kakizaki (GK) rats were chronically implanted with one pair of electrodes in the duodenum. The oral glucose tolerance test (OGTT) and insulin tolerance test (ITT) were performed with or without IES, and plasma glucagon-like peptide-1 (GLP-1) and insulin level were measured. In the other two OGTT sessions, rats were treated with either Exendin (9–39) (GLP-1 antagonist) or Exendin (9–39) plus IES to investigate the underlying mechanism involving GLP-1. Gastric emptying and small intestinal transit were also measured with or without IES. In a chronic study, GK rats were treated with IES or Sham-IES for 8 weeks. Blood glucose, plasma GLP-1 and insulin level, body weight, and food intake were measured. Pancreas weight, islet β-cell apoptosis, and proliferation were also analyzed.

**Results:**

Acute IES reduced blood glucose level from 60 to 120 min during OGTT by 16–20% (all *p* < 0.05, vs. Sham-IES). GLP-1 antagonist significantly blocked the inhibitory effect of IES on hyperglycemia from 15 to 120 min (all *p* < 0.05). IES accelerated the small intestinal transit by 15% (*p* = 0.004). After 8 weeks of chronic stimulation, IES significantly reduced blood glucose (*p* < 0.05) and body weight (*p* = 0.02) and increased the plasma GLP-1 concentration (*p* < 0.05). Furthermore, we observed that chronic IES reduced pancreatic β-cell apoptosis (*p* = 0.045), but showed no effects on β-cell proliferation.

**Conclusions:**

Our study firstly proved the hypoglycemic effect of IES in a rodent model of type 2 diabetes, possibly attributed to the increasing GLP-1 secretion and improvement in β-cell functions.

## Introduction

The estimated total global number of patients with diabetes is 450 million in 2017^1^. Bariatric surgery such as Roux-en-Y gastric bypass (RYGB) is effective in achieving significant long-term weight loss and has been proposed for treating diabetic patients with obesity recently^2^.

Type 2 diabetes (T2D) is the most common type of diabetes characterized with progressive insulin resistance and often associated with obesity. The Goto–Kakizaki (GK) spontaneous diabetic rat is frequently used in basic research for the investigation of mechanisms of diabetes and the development of novel anti-diabetic therapies^3,4^. Main features of the GK rat include decreased β-cell numbers and impaired metabolic functions, reduced glucose-stimulated insulin secretion, glucose intolerance, and chronic inflammation^3,4^.

Although various hypoglycemic medications are available, the current management of diabetes is far from satisfactory due to various side effects and lack of efficacy^5^. One of common complications of hypoglycemic therapy is weight gain, an undesirable outcome with potential long-term adverse consequences on glycemic control itself^6^. Bariatric surgery, such as the RYGB procedure, is the only long-term effective therapy for obesity and has been widely applied for treating morbid obesity^7^.

Bariatric surgery has recently been reported to resolve T2D. Nearly 30% of patients who undergo bariatric surgery have T2D, and for many of them, diabetes resolves after surgery (84 to 98% for bypass procedures)^8,9^. Recent studies have indicated that T2D remission after certain bariatric procedures, such as gastric bypass, can be observed within days after operation, even before any substantial weight loss occurs^10,11^. Accordingly, the accomplishment of rapid glycemic control with gastric bypass is considered to be independent of weight loss although the long-term improvement of glycemic control is certainly related to weight loss^12,13^. While exact mechanisms involved in the hypoglycemic effect of gastric bypass remain elusive, the anatomical alterations of the gastrointestinal tract changes such as: (1) creation of a small gastric pouch reduces meal size, (2) bypass of the proximal small intestine reduces absorption of nutrients, and (3) exclusion of distal stomach inhibits ghrelin; and bypass of the proximal small intestine allows a rapid delivery of nutrients to lower small intestine, resulting in alteration of secretion of gut hormones such as glucagon-like peptide-1 (GLP-1) and glucose-dependent insulinotropic peptide, as well as ghrelin and peptide YY^14–17^. GLP-1 is known to increase glucose-stimulated release of insulin^18–20^ and inhibit release of glucagon. Ghrelin has been reported to be a physiologically negative regulator of insulin release in pancreatic islets and glucose homeostasis^21–23^. The combination of these effects (smaller meal size, reduced nutrient absorption, and increased insulin release due to decreased ghrelin and increased GLP-1) seems to be the basis for the rapid improvement in glycemic control observed after bariatric surgery.

In the present study, we proposed a novel method of intestinal electrical stimulation (IES) for the treatment of diabetes. Previous studies have suggested that IES has the following effects similar to gastric bypass: reduction in rate of gastric emptying, food intake, and nutrient absorption. In preliminary studies, we also found that IES significantly reduced postprandial blood glucose level in rats. Based on these findings, we hypothesized that acute IES reduces postprandial hyperglycemia and that chronic IES improves long-term glycemic control.

The aim of this study was to investigate the hypoglycemic effect of IES and mechanisms involving GLP-1 and preservation of β-cell functions in GK rats, one of the best characterized animal models of spontaneous nonobese T2D.

## Materials and methods

### Animals

Male GK rats at 8 weeks of age (180–200 g) and age-matched Wistar Kyoto (WKY) rats serving as the non-diabetic controls to evaluate the progression of diabetes in GK rats were purchased from Taconic (Germantown, NY, USA). The animals were housed in the climate-controlled facility with a fixed 12-h light and 12-h dark cycle and fed with regular lab chow. The study protocol was approved by the Institutional Animal Care and Use Committee of the VA Medical Center, Oklahoma City, OK, USA.

### Surgery procedure

Under general anesthesia using 2% isoflurane and laparotomy, a pair of stainless steel cardiac pacing wires (Medtronic, Minneapolis, MN, USA) were placed on the seromuscular layer of the duodenum 3 cm beyond the pylorus. The electrode wires were subcutaneously tunneled to the back of the neck, externalized via a silicone and Dacron button (Instech laboratories, Plymouth Meeting, PA, USA), and connected to a tether system. This allowed electrical stimulation to be performed using an external multi-channel stimulator via the tether wires while the animal was housed in its regular cage without any interruption in activity^24^. After the surgical procedure, cefazolin (30 mg/kg) and buprenorphine (0.05 mg/kg were administered for 2 days. None of the experiments were conducted before the animals were completely recovered from the surgery (typically 2 weeks).

### Oral glucose tolerance test

The oral glucose tolerance test (OGTT) was performed as follows: a glucose solution (1 g/kg) was given by oral gavage following an overnight fast. Blood samples were taken via the tail vein before the oral glucose and 15, 30, 60, 120, and 180 min after the glucose administration. The glucose level in blood was determined by a glucometer (Accu-Check Aviva Plus, Roche Diagnostics, Indianapolis, IN, USA). The area under the curve (AUC) of the blood glucose level was calculated using the trapezoidal method.

### Experiment 1: Hypoglycemic effect and mechanisms of acute IES

#### Experiment 1.1: Search for a hypoglycemic method of acute IES

The aim of this experiment was to derive most effective parameters for IES to reduce blood glucose during an OGTT. Twenty 12-week-old male GK rats with one pair of chronic IES electrodes were used to evaluate the hypoglycemic effects of IES with varied parameters in various OGTT sessions with at least a 4-day interval, including a control session and two IES sessions. The IES parameters was set at 3 ms (parameters #1 or IES-3 ms) or 0.3 ms (parameter #2 or IES-0.3 ms), 2 mA, 40 Hz (pulse frequency), and train of time of 0.6 s and off time of 0.9 s. These parameters represent a train frequency of 40 trains/min that is the same as the frequency of the intestinal slow wave in rats. A pulse width of 3 ms was previously shown to alter smooth muscle functions in the experiments of gastric electrical stimulation^25,26^, whereas a pulse width of 0.3 ms was believed to alter only nerve functions^27^. IES was performed during the entire OGTT.

#### Experiment 1.2: Acute IES on insulin sensitivity

An insulin tolerance test (ITT) was conducted to investigate the role of IES on insulin sensitivity. Regular insulin (Humulin U-100; Lilly, Indianapolis, IN, USA) in a saline solution (0.5U/kg) was intraperitoneally injected following a 14 h fast. Blood was collected from the tail vein to measure blood glucose level at baseline and 30, 60, and 120 min after insulin injection. The 12 GK rats with one pair of chronic IES electrodes were studied in two ITT sessions on separate days with IES using the optimized parameters from Experiment 1.1 and Sham-IES (same experimental setup but 0 mA output), respectively.

#### Experiment 1.3: Role of GLP-1 in reducing blood glucose by acute IES

This experiment was to investigate the role of GLP-1 on the inhibitory effect of acute IES on postprandial blood glucose. The same 12 GK rats were studied in four randomized OGTT sessions with following interventions: (1) IES during the entire test session; (2) Sham-IES: same experiment setup for stimulation with 0 mA output; (3) pre-treatment of GLP-1 antagonist, Exendin (9–39) (Phoenix Pharmaceuticals, Inc.) before the OGTT; (4) pre-treatment of Exendin (9–39) before the OGTT and IES during the OGTT. Experimental sessions were at least 4 days apart. Exendin (9–39) (25 nmol/kg, i.p.) was given subcutaneously 30 min before the OGTT^28^. IES was performed during the entire test using the optimized parameters.

#### Experiment 1.4: Effects of acute IES on plasma GLP-1 and insulin

After an overnight fasting, the OGTT was performed in the 12 GK rats using the above-mentioned protocol. The blood samples at different time points were placed in testing tubes containing chilled EDTA, aprotinin, and diprotin A (Sigma-Aldrich, St. Louis, MO, USA) for GLP-1 analysis and in testing tubers with chilled EDTA only for insulin analysis. Plasma GLP-1 was quantified using a commercial ELISA kit (Sigma-Aldrich, St. Louis, MO, USA). A commercial Rat Insulin ELISA kit (EMD Millipore, Temecula, CA, USA) was used to assess the plasma level of insulin.

#### Experiment 1.5: Effects of acute IES on gastric emptying, small bowel transit

Twenty 18-week-old male GK rats were divided into two groups: 10 with optimized IES and another 10 with Sham-IES, the animals were fasted for 18 h with free access to water and then given 10 min to consume 2 g of solid food and finally gavage of 0.5 ml 5% phenol red. After 90 min, the animals were euthanized by inhalation of isoflurane (5%) followed with cervical dislocation. The stomach and whole intestine were removed. The content of the stomach was collected and left in the air to dry for 48 h. Gastric emptying was then determined using the equation: gastric emptying (%)=[1 − (dried gastric content in g/2 g)] × 100^29^. The intestinal transit was measured using an previously established geometric center method as follows: first, the entire length of the small intestine was carefully removed and cut into 10 segments of equal length; then the content of each segment was washed into a testing tube and the amount of the phenol red in each tube was assessed using a spectrophotometer; finally, the geometric center was calculated as described previously^30^.

### Experiment 2: Effects and mechanisms of chronic IES

#### Effects of chronic IES on food intake, body weight, fasting blood glucose, and HbA1c

Twenty GK rats were used to investigate the chronic effects of IES for 2 months on food intake and body weight as well as glycemic profile. After a complete recovery period from the surgical procedure for the placement of electrodes, the animals were kept in individual cages equipped with a BioDAQ continuous food intake monitoring system (Research Diets Inc., New Brunswick, NJ, USA). They were given free access to the food and water^26^. The animals were given a 2-week period for acclimation and then daily food intake was continuously measured by the system for a period of 8 weeks. The GK rats were randomly divided into two groups to receive IES and Sham-IES (wires connected but no stimulation delivered), respectively. IES (40 Hz, 0.6 s on, 0.9 s off, biphasic, 2 mA) was applied during the 12 h in dark (6:00 p.m.–6:00a.m.) using a multi-channel digital pulse generator (World Precision Instrument, Sarasota, FL, USA). Food intake was monitored by a computer via an electronic strain gauge transducer under the food hopper. Body weight and fasting blood glucose were measured once a week over the treatment period. Hemoglobin A1c (HbA1c) was measured before, and 4 and 8 weeks after the interventions.

#### Effects of chronic IES on blood glucose level during OGTT

At baseline and after 4 and 8 weeks of treatment, the GK rats were subjected to an OGTT. Age-matched Wistar rats were subjected to the same OGTT at baseline and 8 weeks afterward. Blood glucose was assessed at baseline, and 15, 30, 60, 120, and 180 min after oral gavage of glucose.

#### Effects of chronic IES on plasma GLP-1 and insulin analysis

At 9 weeks after treatment, under light anesthesia with isoflurance, blood samples were taken from the tail vein at following points: 15 min before, 30 and 60 min after gavage of glucose for GLP-1 and insulin level analysis. The methods were same as in the acute IES experiment.

#### Effects of chronic IES on pancreatic functions

*Fat mass weight*: The subcutaneous, inguinal, and visceral fat depots (retroperitoneal, epididymal, and mesenteric) were quickly removed after the rats were sacrificed. The tissues were dissected, weighed, and summed to provide a measurement of body fat. The adiposity index is calculated as 100% × (sum of fat pad weights)/(body weight)(% FM/BW)^31^ and100% × (sum of visceral fat pad weights)/(body weight)(% VFM/BW).

#### TUNEL staining for pancreatic cell apoptosis

After euthanization, the pancreas was excised and weighed from Wistar rats, GK rats with Sham-IES, and GK rats with IES (*n* = 5 in each group). The relative pancreas weight was expressed as 100% × (pancreas weight)/(body weight). After fixation in 10% neutral-buffered formalin (NBF) for 24 h, tissues were paraffin embedded. A 6μm paraffin section was analyzed for fragmented DNA in apoptotic cells using the terminal deoxynucleotidyl transferase UTP nick-end labeling (TUNEL) technique and a Millipore ApopTag plus peroxidase in situ apoptosis detection kit (EMD Millipore, Temecula, CA, USA) as follows: after dewaxing and two times of 5-min wash in sterile water, a solution of proteinase K (20 µg/ml) was added to the section and incubated for 15 min at room temperature. Specimens were then washed two times in a PBS solution and between consecutive stages. Endogenous peroxidase was quenched in 3.0% hydrogen peroxide for 5 min at room temperature. After 10 s exposure to equilibration buffer (supplied in the ApopTag Peroxidase Kit), a TdT enzyme was added onto the sections and incubated in a humidified chamber at 37 °C for 1 h. After washout, an anti-digoxigenin conjugate was then added and incubated in a humidified chamber at 37 °C for 30 min. Finally, a diaminobenzidine solution was added; 5 min later, the section was washed for 2 min under running tap water; the section was then counterstained with haematoxylin and 1 min later rinsed under running tap water for 1 min. We used the ImageJ software for the measurement of the area of islet. The TUNEL-positive cell was manually counted by an experimenter blind to the treatment and normalized by the area of islet.

*Cell proliferation in pancreas islet*: Six hours before the pancreas removal, rats were administered with bromodeoxyuridine (BrdU) solution at a dose of 100 mg/kg by intraperitoneal injection. After euthanization, the pancreas was excised and weighed from Wistar rats, GK rats with Sham-IES, and GK rats with IES (*n* = 5 in each group). After fixation in 10% NBF for 24 h, tissues were paraffin embedded. A 6μm paraffin section from each case was analyzed for the BrdU mark for the detection of proliferating cells in the pancreas cells using a BrdU staining kit (Abcam, CA, USA), according to the manufacturer’s instructions.

### Statistics and analysis

Data are expressed as mean ± SE. The independent-sample Student’s *t* test was used to compare the difference between the Sham and IES groups. The non-normal distribution data were analyzed by Mann–Whitney *U* test. Two-way repeated measurement of analysis of variance was applied to investigate the difference in blood glucose, plasma GLP-1, and insulin among different groups. The post hoc Fisher's test was used to compare the difference between two groups. *P* value of <0.05 was considered significant.

## Results

### Optimized IES acutely improved the glucose tolerance in GK rats

**Fig. 1:**
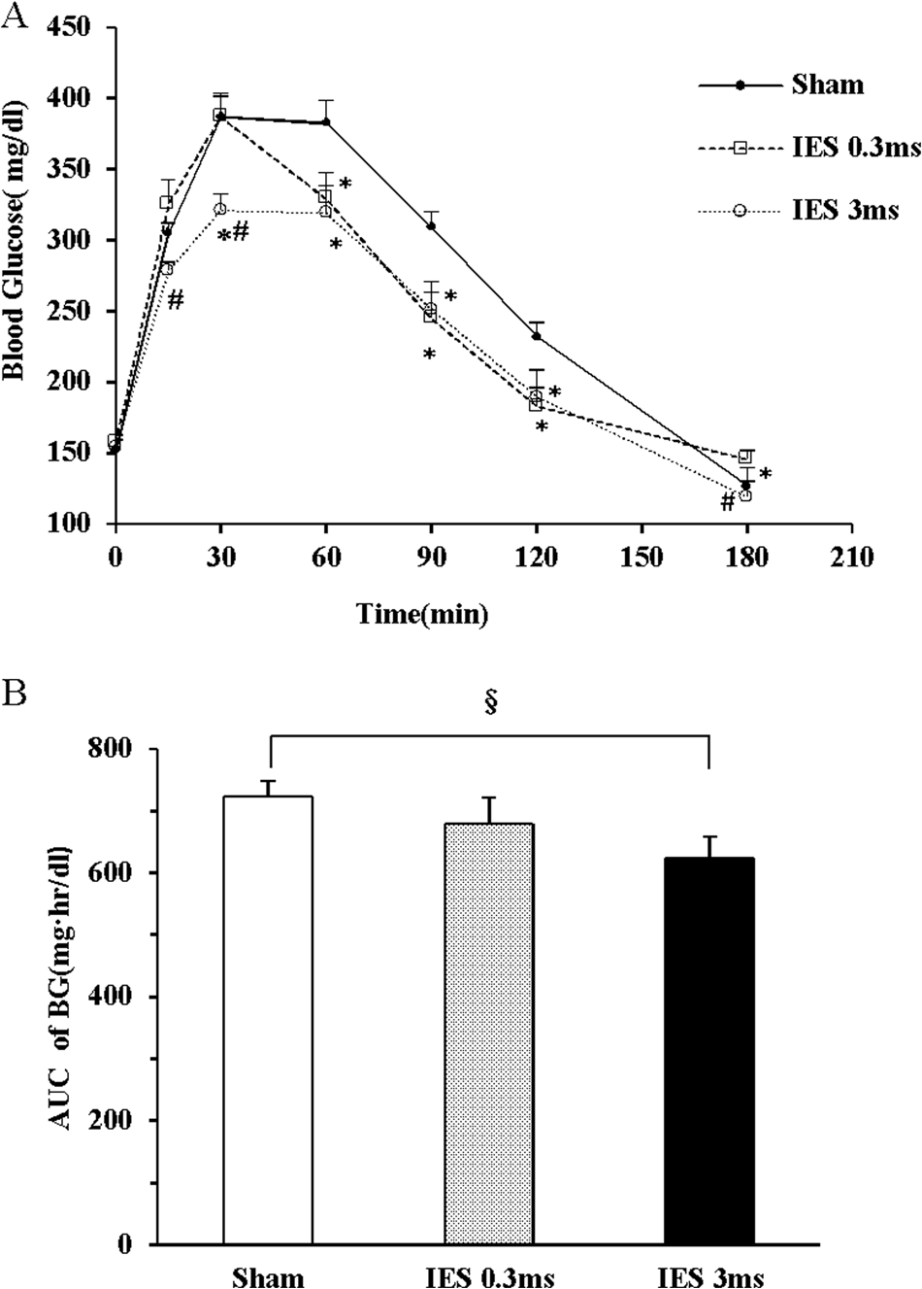
Effects of acute IES on blood glucose with different parameters. **a** Blood glucose during OGTT. **b** AUC of blood glucose (*p < 0.05 vs. Sham; ^#^*p* < 0.05 vs. IES-0.3 ms; ^§^*p* < 0.01 vs. Sham). IES, intestinal electrical stimulation; OGTT, oral glucose tolerance test; AUC, area under curve; BG, blood glucose

IES with different pulse widths were examined if they had hypoglycemic effects during OGTT. At 30 min after gavage of glucose, IES of 3 ms reduced blood glucose to 321 ± 19 mg/dl compared to 388 ± 16 mg/dl (*p* = 0.013) with Sham-IES and 387 ± 16 mg/dl (*p* = 0.014) with IES of 0.3 ms. Then after 30 min, both IES- 3ms and IES-0.3 ms reduced blood glucose by 16–20% (all *p* < 0.05) and no difference was noted between these two parameters (Fig. 1a). Compared to Sham, IES of 3 ms significantly reduced the postprandial AUC for blood glucose (*p* = 0.004), while IES of 0.3 ms had a marginal effect (*p* = 0.063) (Fig. 1b).

### Effects of Exendin (9–39) on blood glucose

**Fig. 2:**
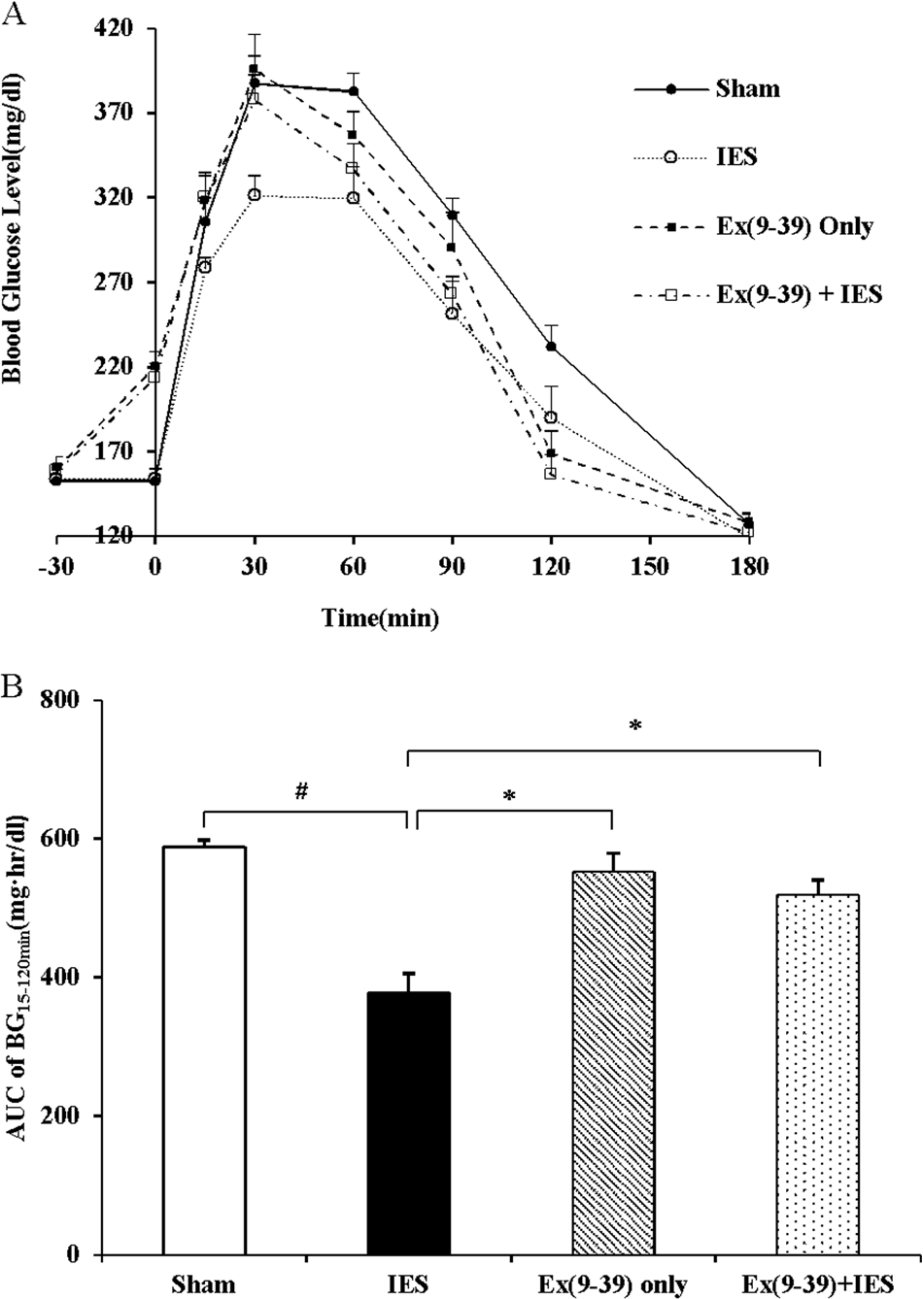
Inhibitory effect of GLP-1 antagonist on IES. **a** Blood glucose during OGTT: 0 min, Ex (9–39)/Ex (9–39) + IES vs. Sham or IES, *p* = 0.000; 15 min, Ex (9–39)/Ex (9–39) + IES vs. IES,  *p*< 0.05; 30 min, Ex (9–39)/Ex (9–39) + IES /Sham vs. IES, *p* < 0.05; 60 min and 90 min, Ex (9–39)/Ex (9–39) + IES /Sham vs. IES, *p* < 0.05, Ex (9–39) + IES vs. Sham, *p* < 0.05; 120 min, Ex (9–39)/Sham vs. IES, *p* < 0.05, Ex (9–39)/Ex (9–39) + IES vs. Sham, *p* < 0.01. **b** Area under curve of blood glucose during OGTT (15–120 min), Ex (9–39)/Ex (9–39) + IES /Sham vs. IES, *p < 0.05, ^#^*p* < 0.01. GLP-1, glucagon-like peptide-1; IES, intestinal electrical stimulation; OGTT, oral glucose tolerance test; AUC, area under curve; BG, blood glucose; ex (9–39), Exendin (9–39), GLP-1 antagonist

The effect of Exendin (9–39), an antagonist of the GLP-1 receptor, on the ability of IES to lower blood glucose during an OGTT also was examined (Fig. 2a, b). As shown in Fig. 2a, Exendin (9–39) increased the fasting blood glucose level to 220 ± 9 mg/dl from a baseline of 160 ± 7 mg/dl [Ex (9–39)/Ex (9–39) + IES vs. Sham or IES, *p* = 0.000], but significantly blocked the inhibitory effect of IES on hyperglycemia from 15 to 120 min [Ex (9–39)/Ex (9–39) + IES vs. IES, all *p* < 0.05]. As shown in Fig. 2b, Exendin (9–39) had significant antagonism effect on the inhibitory effect of IES [Ex (9–39)/Ex (9–39) + IES vs. IES, both *p* < 0.05] on the AUC of blood glucose during the OGTT (15–120 min).

### Acute IES increased the blunted postprandial GLP-1 secretion in GK rats

**Fig. 3:**
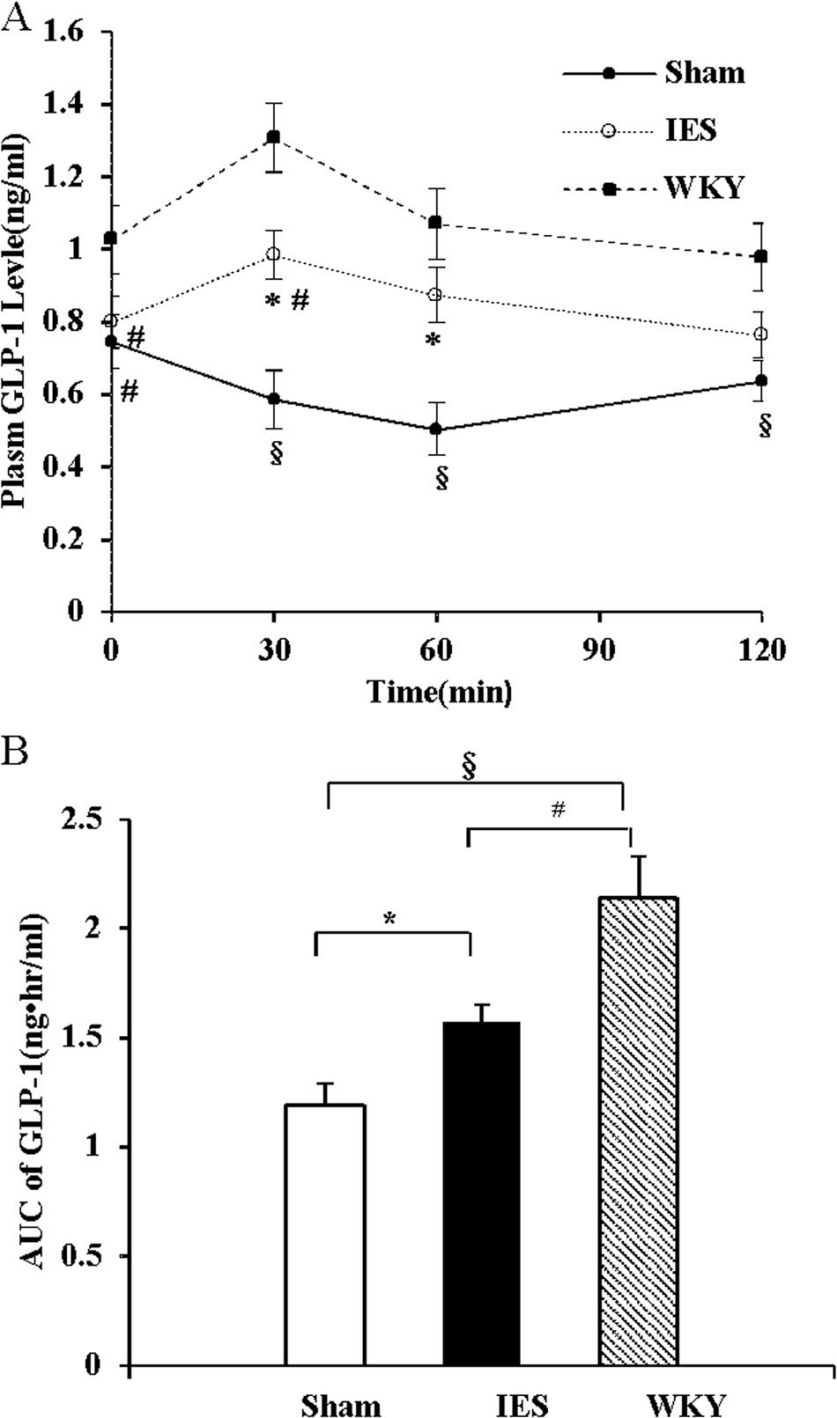
Acute IES increase GLP-1 secretion. **a** Plasma GLP-1 level during OGTT. **b** AUC of plasma GLP-1 level during OGTT (*p < 0.05 vs. Sham; ^#^*p* < 0.05 vs. WKY; ^§^*p* < 0.01 vs. WKY). IES, intestinal electrical stimulation; OGTT, oral glucose tolerance test; GLP-1, glucagon-like peptide-1; AUC, area under curve

The fasting plasma GLP-1 concentration was higher in WKY rats than that in GK rats. After glucose administration, the plasma GLP-1 concentration increased at 30 min and returned to the baseline level 60 min later in WKY rats, while that in GK rats decreased 30 min later. IES increased plasma GLP-1 concentrations by 66% (*p* < 0.01, vs. Sham) at 30 min and by 70% (*p* < 0.05, vs. Sham) at 60 min after glucose administration. Acute IES increased the AUC of plasma GLP-1 by 32% (*p* < 0.05, vs. Sham) (Fig. 3a, b).

### Acute IES increased early phase insulin secretion in GK rats

Acute IES improved insulin secretion at first 60 min during the OGTT; at 30 min, the insulin level was increased by 37 % (*p* < 0.05, vs. Sham). Thereafter, the insulin level was decreased rapidly and no difference was found in AUC of insulin between two groups. IES showed no significant difference in comparison with Sham-IES on the absolute value and the percentage of blood glucose reduction during the ITT (Supplementary Information).

### Acute IES accelerated intestinal transit

Gastric emptying in the GK rats was delayed compared to WKY rats (48 ± 9.0% vs. 58.3 ± 15.9%, *p* < 0.05). IES showed no effect on gastric emptying (49.0 ± 12.9% vs. 48 ± 9.0%, *p* > 0.5 vs. Sham). However, IES accelerated small intestinal transit represented as the geometric center (7.2 ± 0.7 vs. 6.2 ± 0.6, vs. Sham, *p* = 0.004).

### Effects of chronic IES on fasting glucose, HbA1c

**Fig. 4:**
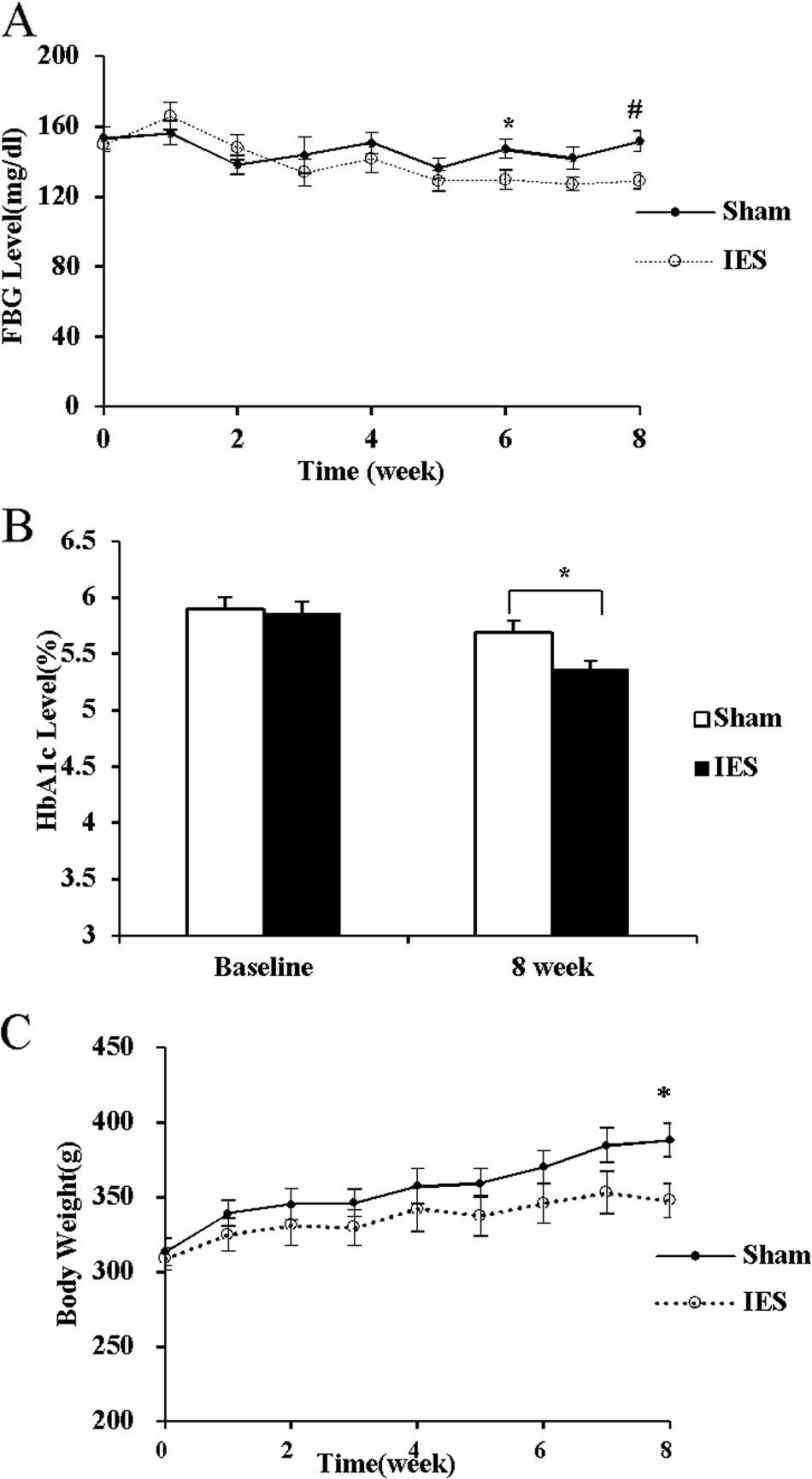
Chronic IES on blood gluse and body weight. Chronic IES on fasting blood glucose **a** HbA1c **b**, and body weight **c**. (*p < 0.05, ^#^*p* < 0.01 vs. Sham). IES, intestinal electrical stimulation; HbA1c, hemoglobin A1c; FBG, fasting blood glucose; BW: body weight

Before the 8-week interventions, fasting plasma glucose concentrations were similar in the two groups of GK rats, which was greater (177 ± 6 mg/dl) than that in their age-matched WKY control rats (98 ± 4 mg/dl, *p* < 0.001). Chronic IES treatment decreased fasting plasma glucose at the end of the 8-week treatment. The fasting glucose concentration was 12 and 15% lower at the sixth-week (*p* < 0.05) and the eighth-week intervention (*p* < 0.01), respectively, compared to that in GK rats with Sham-IES (Fig. 4a). After the 8-week interventions, the GK rats with IES had a lower HbA1c level than those with Sham-IES (*p* < 0.05) (Fig. 4b).

### Effects of chronic IES on food intake, body weight and adipose index

Chronic IES showed no effects on the weekly food intake and the cumulative food intake at the first and second month, but decreased the weight gain in the GK rats at the end of 8-week treatment: the final body weight was 348 ± 11 g in the IES group, but 388 ± 11 g in the Sham group (*p* = 0.02, Fig. 4c). Moreover, the GK rats with IES showed a significant lower adipose index than those with Sham-IES (4.2 ± 0.4% vs. 5.8 ± 0.4%, *p* = 0.018). IES reduced visceral fat mass nearly 30% compared to Sham-IES (2.2 ± 0.17% vs. 3.0 ± 0.25%, *p* < 0.05).

### Chronic IES improved impaired glucose intolerance in GK rats

**Fig. 5:**
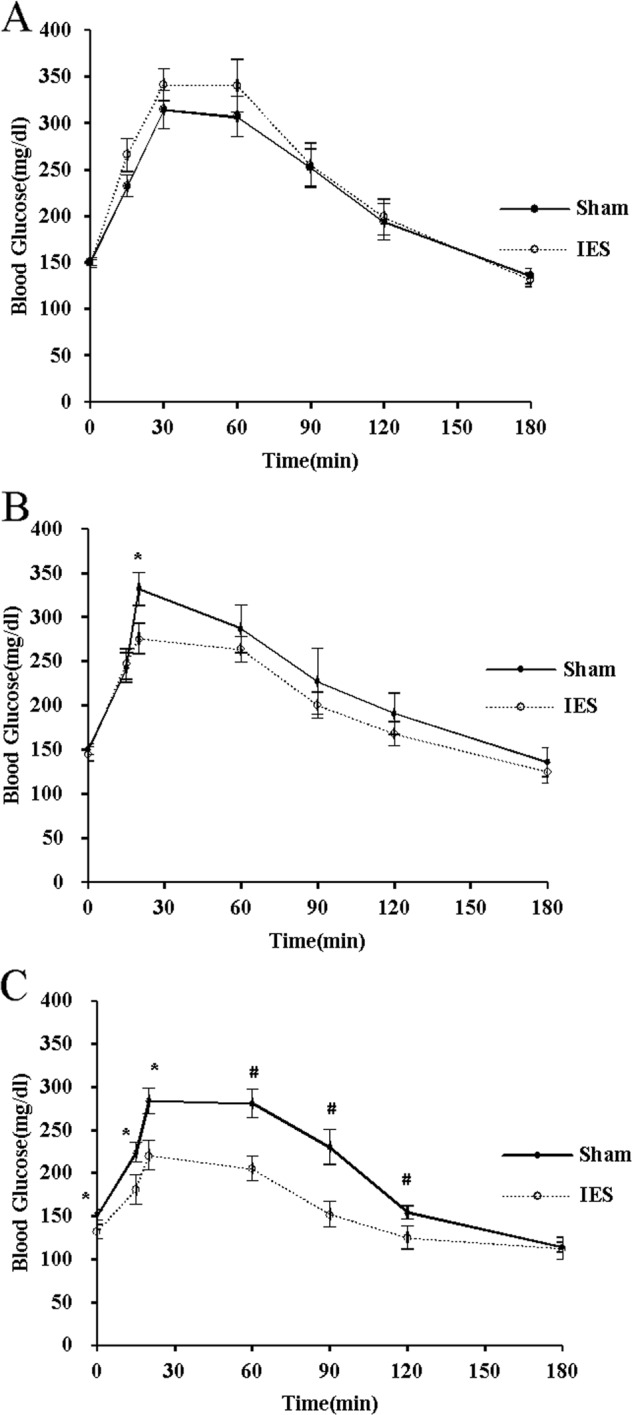
Chronic IES on glucose intolerance. **a** Baseline, **b** the fourth week, **c** the eighth week, **d** AUC of blood glucose (*p < 0.05, ^#^*p* < 0.01 vs. Sham). IES, intestinal electrical stimulation; AUC, area under curve

At the commence of this study, no difference was found between the blood glucose and AUC for blood glucose between two groups of GK rats, though they had significantly greater fasting and postprandial blood glucose levels than their age-matched WKY rats (Fig. 5a). As shown in Fig. 5b, at 4 weeks, chronic IES reduced the blood glucose level significantly at 30 min (*p* < 0.05). As shown in Fig. 5c, at 8 weeks, chronic IES reduced the increased blood glucose level by 20–30% from 15 to 120 min (*p* < 0.05 at 15 and 30 min, *p* < 0.01 at 60 and 120 min). Chronic IES decreased the AUC of blood glucose (*p* = 0.002, vs. baseline), while no effect was observed in GK rats with Sham-IES.

### Chronic IES on insulin secretion and insulin sensitivity

**Fig. 6:**
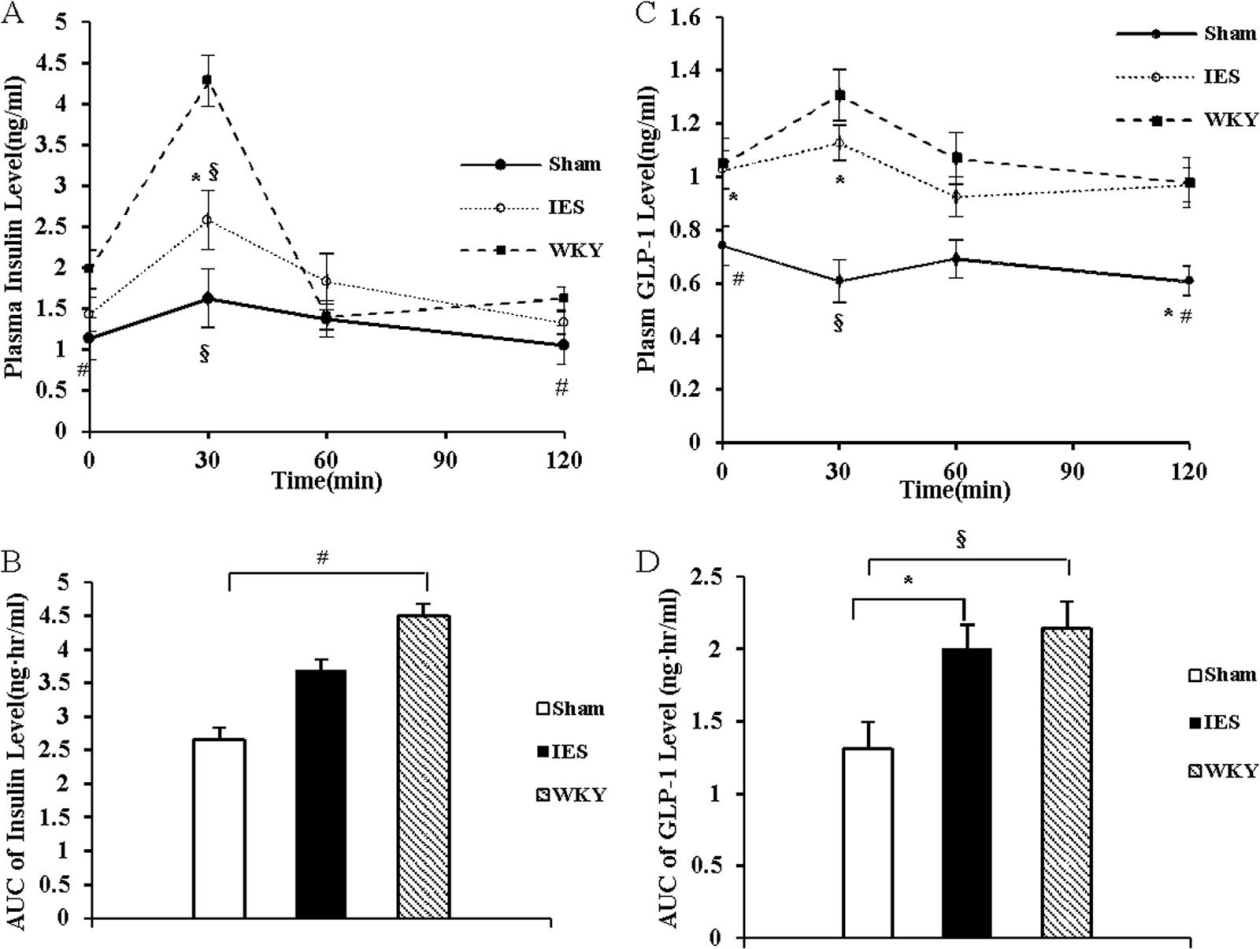
Chronic IES on insulin secretion and GLP-1 level during OGTT. **a** Insulin level; **b** AUC of insulin; **c**GLP-1 level; **d** AUC of GLP-1 (*p < 0.05 vs. Sham; ^#^*p* < 0.05, ^§^*p* < 0.01 vs. WKY). IES, intestinal electrical stimulation; OGTT, oral glucose tolerance test; GLP-1, glucagon-like peptide-1; AUC, area under curve; GLP-1, glucagon-like peptide-1

Compared with the WKY control rats, the insulin level was lower at all time points except 60 min in the GK rats with Sham-IES, but was lower only at 30 min in the GK rats with IES (Fig. 6a). Chronic IES increased insulin level at 30 min (*p* < 0.05), no difference was noted at other time and AUC (Fig. 6a, b). IES did not change blood glucose level after insulin injection during ITT, which suggested that may be chronic IES did not change the insulin sensitivity (see Supplementary Information).

### Chronic IES on GLP-1 secretion

IES increased fasting GLP-1 secretion by 38% (*p* < 0.05, vs. Sham). IES increased GLP-1 by 84% at 30 min after glucose administration (*p* < 0.05, vs. Sham, Fig. 6c) and the AUC of GLP-1 by 53% (*p* < 0.05, vs. Sham, Fig. 6d). The GLP-1 level in the GK rats after chronic IES was comparable to the control WKY rats, suggesting that chronic IES almost normalized GLP-1 secretion in the diabetic rats.

### Effects of chronic IES on β-cell apoptosis and proliferation

The pancreas weight was lower in the GK rats treated with Sham-IES (0.21 ± 0.02% of the total body weight) than that in the control rats (0.28 ± 0.02%, *p* = 0.041). Chronic IES increased the relative pancreas weight by 0.26 ± 0.02% (*p* = 0.012, vs. Sham).

Compared to very few apoptotic cells found in the pancreas islets in WKY rats (0.233 ± 0.119/10,000 µm^2^), the number of apoptotic cells in pancreas islet was significantly increased to 2.631 ± 0.245/10,000 µm^2^ in the GK rats with Sham-IES (*p* < 0.001, vs. WKY). The chronic IES reduced apoptosis 1.736 ± 0.421/10,000 µm^2^ (*p* = 0.045, vs. Sham). However, chronic IES showed no effects on cell proliferation (see Supplementary Information).

## Discussion

T2D involves complex pathophysiological mechanisms, making treatment difficult. Common treatments, including lifestyle intervention and medications, are beyond optimal and often unsatisfactory. Maintaining long-term stability of blood glucose remains a major challenge. In recent years, metabolic surgery has been applied in obese patients with T2D and has shown promising results: weight loss, improvement in glucose metabolism, and even complete cure of diabetes^8^. It has been shown that its hypoglycemic effect is independent of weight loss^12,13^ and may be related to changes in gastrointestinal hormones, such as GLP-1^14^. However, it is limited in clinical applications due to its invasive nature and non-reversible changes in anatomy of the gut as well as complications.

IES was initially introduced for the treatment of motility disorders, such as pseudo-intestinal obstruction, postoperative ileus, and other gastrointestinal motility diseases. Recently, it has been reported that IES can delay gastric emptying^32^, accelerate intestinal transit^30,33,34^, and reduce food intake and nutrient absorption in animal models. These gastrointestinal motility changes in rats and pigs result in food intake reduction and weight loss^32,35^. In addition, IES can decrease ghrelin in the stomach^36^ and increase GLP-1 secretion in rats^37,38^. These findings are very similar to those observed after bariatric surgery, suggesting a potential of IES for treating diabetes. However, compared with bariatric surgery, IES is less invasive, adjustable, reversible, and easier to implement.

Very few studies reported the effect of IES on blood glucose. Khawaled et al.^39^ reported that duodenal electrical stimulation decreased blood glucose in normal rats during an OGTT. The stimulation decreased the amplitude and peak of blood glucose level after glucose administration in 33 male SD rats, and the AUC of blood glucose by about 50%^39^. IES via ring electrodes attached to a nasogastric tube in the duodenum (10 times/min, 300 ms, 5 mA) decreased blood glucose level from 15 to 90 min after 150 g of glucose loading in 10 healthy volunteers^40^. However, it was unknown whether IES would have a hypoglycemic effect in animal models of diabetes or patients with diabetes.

To the best of our knowledge, this was the first study to explore and prove the hypoglycemic effect of IES on T2D animal model. We have tried two methods of IES: IES of 3 ms and IES of 0.3 ms and found that IES of 3 ms was more potent than IES of 0.3 ms. Since IES of 3 ms is known to alter intestinal transit, whereas IES of 0.3 ms is unable to alter gastrointestinal motility^24^, these findings seemed to suggest that alteration in gastrointestinal motility played an important role in the hypoglycemic effect of IES.

The GK rats, one of the best characterized animal models of spontaneous T2D^3,4^, were used in this study. The GK line was established by repeated inbreeding from Wistar rats selected at the upper limit of normal distribution for glucose tolerance and the GK rat has become a stable model of T2D and exhibits hyperglycemia, insulin resistance, hyperinsulinemia, and a number of pancreas islet β-cell dysfunctions. It is the most widely used model of T2D and has also been frequently used in studying the neuroendocrine mechanisms involved in the metabolic effects of bariatric surgery^41,42^. In the present study, we proved the hypoglycemic effect of IES on this animal model effectively, but IES did not improve insulin sensitivity in the ITT, which might be attributed to the fact that the GK rats was lean and might not exhibit serious insulin resistance. Glucose uptake experiments should be done to confirm this conclusion in the future study.

To explore the possible underlying mechanisms involving gastrointestinal motility, we studied the effects of IES on gastric emptying and intestinal transit. While intestinal transit was accelerated as expected, gastric emptying was surprisingly not delayed by IES. In previous studies that IES was found to delay gastric emptying in normal and obese animals. The discrepancy might be attributed to the fact that the GK rats used in this study had delayed gastric emptying already in comparison with the normal control rats. We speculated that the IES-induced acceleration in intestinal transit might play a role in the enhanced release of GLP-1 by bringing more nutrients to the distal end of the small intestine.

The role of GLP-1 in the hypoglycemic effect of IES was indicated in this study. Exendin (9–39) partly blocked the hypoglycemic effect of IES, indicating the involvement of GLP-1 pathway. GLP-1 is a neuropeptide that is secreted by L cells in the distal segment of the small intestine in a nutrient-dependent manner^43^, which regulates blood glucose homeostasis^44,45^ by altering insulin secretion and also affects glucose transport through a non-pancreatic-dependent mechanism^46,47^. The importance of GLP-1 in the regulation of blood glucose has been confirmed by many clinical and basic studies; GLP-1 has been used for the treatment of type 2 diabetes. It has been confirmed in vivo studies that electrical stimulation can enhance GLP-1 secretion^37^. Direct stimulation of the isolated distal ileumin vitro increased nutrition-induced GLP-1 secretion. Sandoval et al.^38^ found that IES increased the GLP-1 level at the presence of intraluminal nutrients. In the present study, we noted that there was an absence of postprandial surge in the GK rats treated with Sham-IES, but a normal postprandial increase in the GK rats treated with IES, a pattern similar to that in the normal control rats.

The intestinal insulinotropic effect of GLP-1 is known responsible for over 50% of postprandial insulin secretion. Consistent with IES-induced increase in GLP-1, IES also increased insulin secretion at 30 min after glucose administration; however, the increase was only at that particular time, suggesting that IES improved the early-phase insulin secretion, but not the second-phase insulin secretion. IES did not induce hyperinsulinemia, but reduced the risk of pre-prandial hypoglycemia. In contrast, Khawaled et al.^39^ reported that IES decreased insulin secretion by 21% mostly during the first 30 min after glucose load. In a human volunteer study, Liu et al.^40^ observed that the insulin level in the IES session at 30 min was a little lower than the Sham-IES session (*p* = 0.06). However, these studies were conducted in normal rats and humans in which the insulin secretion pattern is known to be completely different from the diabetes. In diabetes, the early-phase insulin secretion is deficient or absent, whereas the second-phase insulin secretion is delayed and exaggerated.

We also investigated the chronic hypoglycemic effects of IES on blood glucose in the GK rats. At the end of 8 weeks of continuous treatment, there was a significant difference in blood glucose during the OGTT between the IES group and Sham group. More encouragingly, chronic IES also reduced fasting blood glucose by 10 to 15%. This finding was beyond our expectation since IES was designed to reduce postprandial blood glucose; however, it proved our hypothesis that chronic IES might improve fasting blood glucose by preserving pancreatic and β-cell functions. Histological analyses confirmed our hypothesis. The ratio of pancreas weight and body weight in Sham GK rats was significantly lower than that of the control rats; however, the ratio in the GK rats with chronic IES was comparable to the normal control rats, suggesting that chronic IES prevented the loss of the pancreatic mass. The immunohistochemical analysis of islets showed that the islet cell apoptosis was significantly reduced with IES in comparison with Sham-IES.

Chronic IES improved GLP-1 secretion to a level comparable to the normal rats. In the acute IES study, we found that acute IES could increase peak GLP-1 secretion after glucose load, but the GLP-1 secretion during the entire OGTT was still lower than that in normal rats. With chronic IES, however, the GLP-1 level during the OGTT was not significantly different from the control WKY rats. GLP-1 has been shown to improve glucose-dependent insulin secretion^18,48^ in many studies, reduce glucagon level, improve islet cell proliferation and apoptosis, and the ratio of α and β cell^19,49^. Therefore, we hypothesized that the improvement of islet morphology might be attributed to the IES-induced improvement in GLP-1 secretion.

Chronic IES reduced body weight by about 10% and the whole-body fat mass, especially visceral fat at the end of study. As the GK rats were lean, the weight loss by chronic IES was not dramatic and the effect of IES on insulin resistance was not noted.

In conclusion, acute IES improves postprandial blood glucose in GK rats, possibly mediated via its accelerative effect on intestinal transit and the improvement in GLP-1 secretion and early-phase insulin secretion. Chronic IES improves both fasting and postprandial blood glucose and the improved fasting blood glucose might be attributed to the preserving effects of IES on pancreatic and islet cell functions.

## Supplementary information

Supplement 1

Supplement 2

Supplement 3
