# Supplementary figures and images for: Intestinal electrical stimulation attenuates hyperglycemia and prevents loss of pancreatic β cells in type 2 diabetic Goto–Kakizaki rats
Source: Nutr Diabetes. 2019 Feb 6;9:4. doi: 10.1038/s41387-019-0072-2 (PMC6365494; doi:10.1038/s41387-019-0072-2)

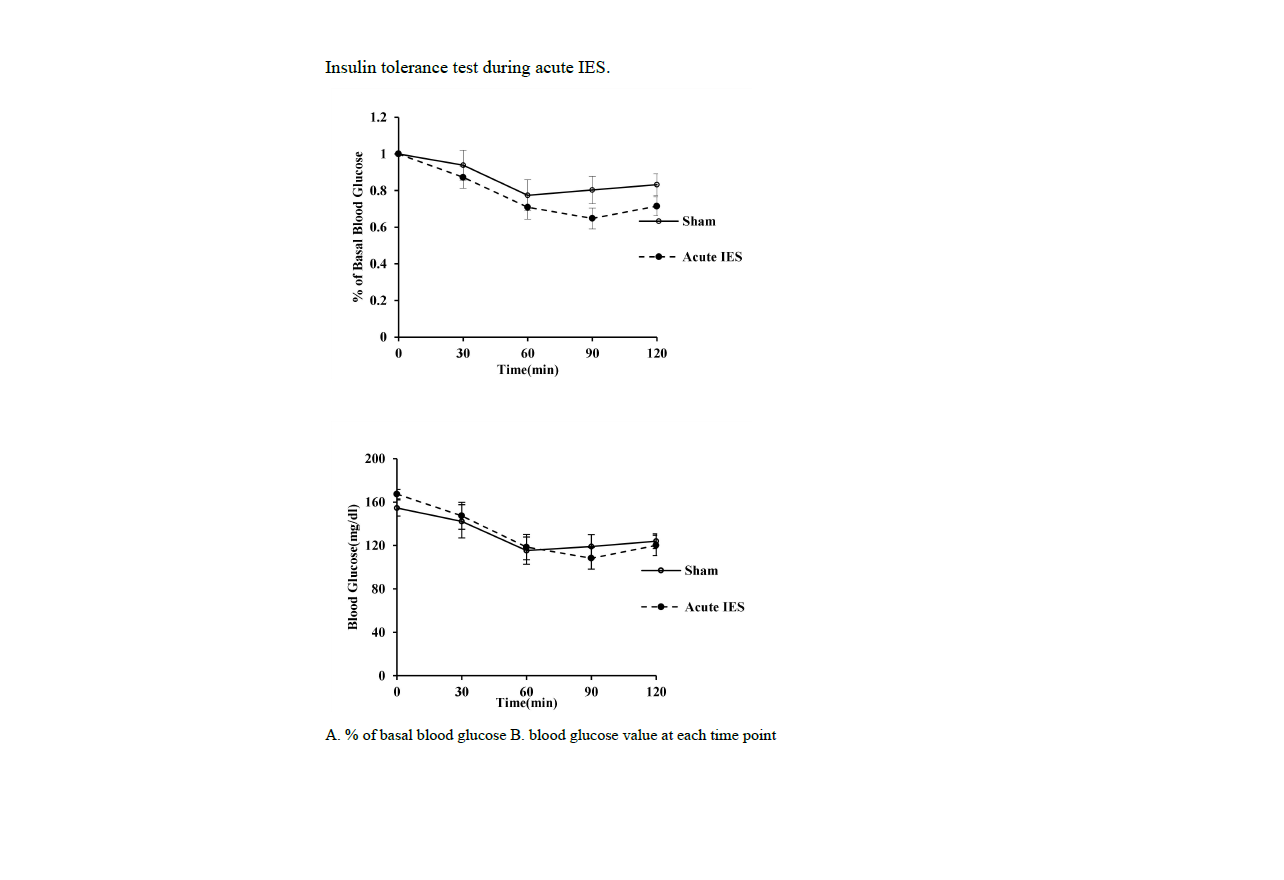

Supplement: Supplementary file 1 — Supplement 1 [file 41387_2019_72_MOESM1_ESM.tif]

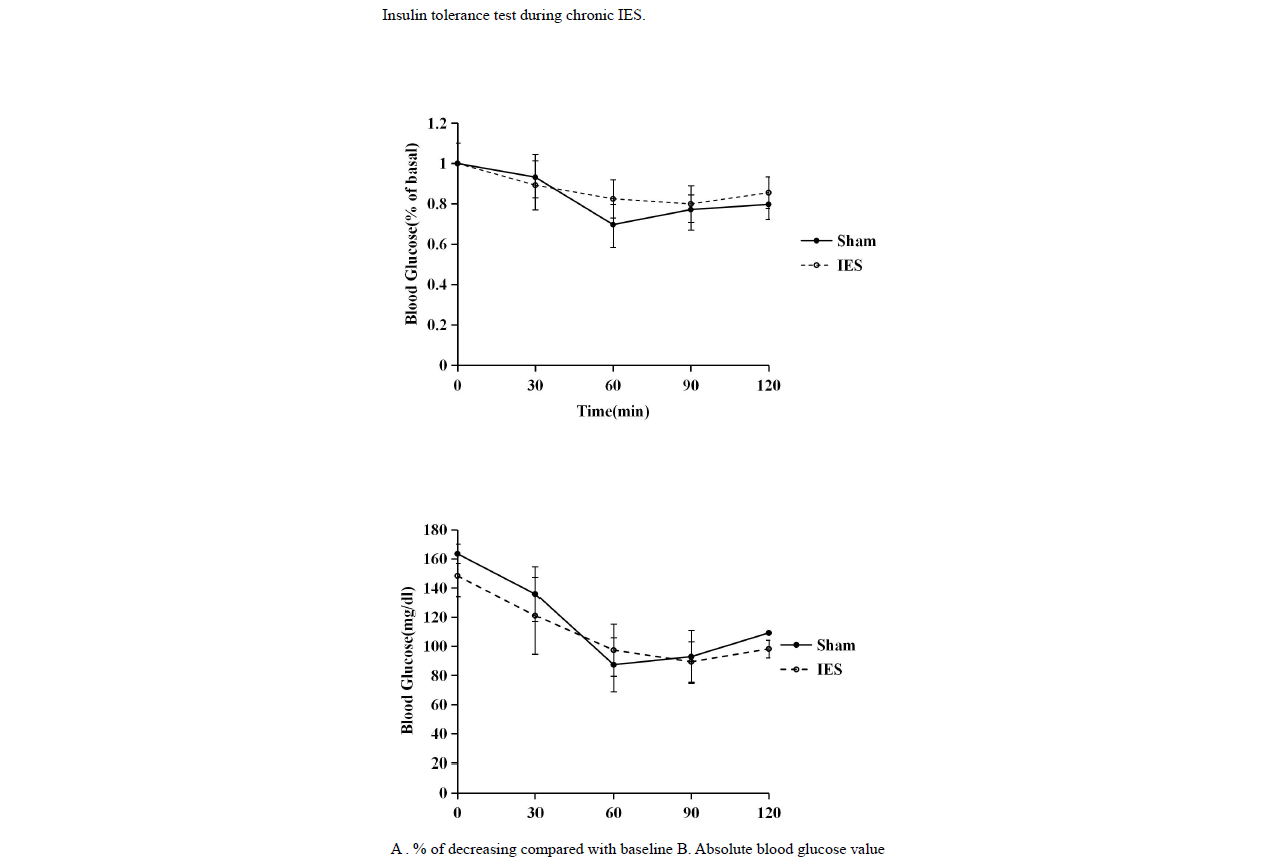

Supplement: Supplementary file 2 — Supplement 2 [file 41387_2019_72_MOESM2_ESM.tif]

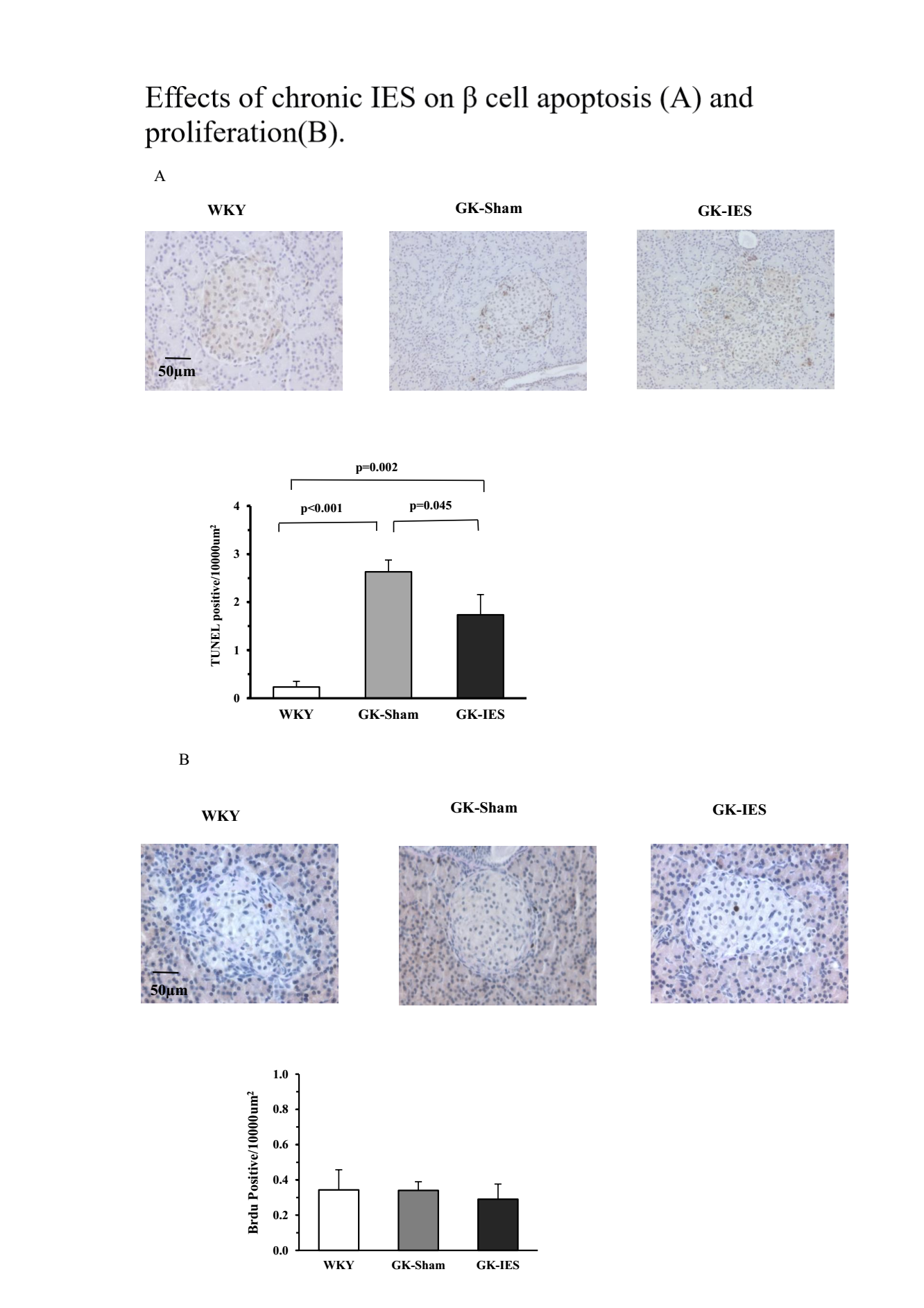

Supplement: Supplementary file 3 — Supplement 3 [file 41387_2019_72_MOESM3_ESM.tif]
